# Co-expression analysis identifies long noncoding RNA *SNHG1* as a novel predictor for event-free survival in neuroblastoma

**DOI:** 10.18632/oncotarget.11158

**Published:** 2016-08-09

**Authors:** Divya Sahu, Chia-Lang Hsu, Chen-Ching Lin, Tz-Wen Yang, Wen-Ming Hsu, Shinn-Ying Ho, Hsueh-Fen Juan, Hsuan-Cheng Huang

**Affiliations:** ^1^ Institute of Bioinformatics and Systems Biology, National Chiao Tung University, Hsinchu, Taiwan; ^2^ Bioinformatics Program, Taiwan International Graduate Program, Institute of Information Science, Academia Sinica, Taipei, Taiwan; ^3^ Institute of Biomedical Informatics, Center for Systems and Synthetic Biology, National Yang-Ming University, Taipei, Taiwan; ^4^ Department of Life Science, National Taiwan University, Taipei, Taiwan; ^5^ Institute of Molecular and Cellular Biology, National Taiwan University, Taipei, Taiwan; ^6^ Department of Surgery, National Taiwan University Hospital, Taipei, Taiwan; ^7^ Department of Biological Science and Technology, National Chiao Tung University, Hsinchu, Taiwan; ^8^ Graduate Institute of Biomedical Electronics and Bioinformatics, National Taiwan University, Taipei, Taiwan

**Keywords:** neuroblastoma, long noncoding RNAs, SNHG1, co-expression study, event-free survival

## Abstract

Despite of the discovery of protein therapeutic targets and advancement in multimodal therapy, the survival chance of high-risk neuroblastoma (NB) patients is still less than 50%. *MYCN* amplification is a potent driver of NB, which exerts its oncogenic activity through either activating or inhibiting the transcription of target genes. Recently, long noncoding RNAs (lncRNAs) are reported to be altered in cancers including NB. However, lncRNAs that are altered by *MYCN* amplification and associated with outcome in high-risk NB patients are limitedly discovered. Herein, we examined the expression profiles of lncRNAs and protein-coding genes between *MYCN* amplified and *MYCN* non-amplified NB from microarray (*n* = 47) and RNA-seq datasets (*n* = 493). We identified 6 lncRNAs in common that were differentially expressed (adjusted *P* ≤ 0.05 and fold change ≥ 2) and subsequently validated by RT-qPCR. The co-expression analysis reveals lncRNA, *SNHG1* and coding gene, *TAF1D* highly co-expressed in NB. Kaplan-Meier analysis shows that higher expression of *SNHG1* is significantly associated with poor patient survival. Importantly, multivariate analysis confirms high expression of *SNHG1* as an independent prognostic marker for event-free survival (EFS) (HR = 1.58, *P* = 2.36E-02). In conclusion, our study unveils that *SNHG1* is up-regulated by *MYCN* amplification and could be a potential prognostic biomarker for high-risk NB intervention.

## INTRODUCTION

Neuroblastoma (NB) is a cancer of undifferentiated sympathetic neuroblasts that accounts for approximately 10% of all childhood cancer worldwide [1, 2]. It frequently originates in adrenal medulla but can develop in the sympathetic ganglia of the chest, abdomen or pelvis [1–3]. The tumor either regress spontaneously in infants or undergo relentless proliferation in children older than 1 year of age, and is characterized by the patient's age at diagnosis, spread of the disease or genetic heterogeneity led by chromosomal aberration, oncogene amplification or allelic loss [1–3]. These diverse clinical presentations stratify NB tumors into risk groups in which low-risk group has a good prognosis and is cured by surgery alone, but high-risk group has a very poor prognosis despite of intensive chemotherapy [3–5]. Genome-wide surveys have identified a large number of protein biomarkers [6, 7], among which v-myc avian myelocytomatosis viral oncogene neuroblastoma derived homolog (*MYCN*) oncogene, is a strong prognostic marker for advance stage NB, indicating a poor survival rate [8–10]. Additionally, anaplastic lymphoma kinase (*ALK*) oncogene, which is amplified in 3–4% and frequently mutated in 6–10% of NB cases, reported to be another promising target for the disease treatment [11–14]. Therapeutic strategies such as targeting neuroblastoma cell surface disialoganglioside GD2 antigen with monoclonal antibodies has also shown a substantial improvement in the patient outcome [15]. Although, protein biomarkers and multimodal therapies have improved NB patients survival, still the survival chance for event-free survival (EFS) in high-risk group is less than 50% [5]. Therefore, there is a need to find new crucial players at the molecular level that can significantly associate with NB prognosis.

With advancement in the field of molecular biology and high-throughput techniques, it is now known that large portions of the mammalian genome are noncoding, spanning from short RNAs (including miRNAs, piRNAs, snoRNAs) to long RNAs of transcript length greater than 200 nucleotides [16, 17]. Long noncoding RNAs (lncRNAs), are regulatory RNAs transcribed either from intragenic or intergenic locus of the genome and constitute major proportion of the cellular transcripts [18–20]. The growing number of evidences show involvement of lncRNAs in gene-regulation, transport, differentiation, dosage-compensation, and protein synthesis [21–24]. Studies have also pointed out their expression to be altered in various types of cancer including NB, initiating tumor development and progression [22, 25–27]. For instance, the expression of lncRNA, neuroblastoma associated transcript-1 (*NBAT-1*) is down-regulated in high-risk NB and associated with poor patient survival [28]. Loss of *NBAT-1* contributes to aggressive NB, indicating its importance as a tumor suppressor lncRNA [28]. The expression of lncRNA, short *CASC15* isoform (*CASC15-S*) is also down-regulated in high-risk NB promoting neural growth and differentiation [29]. The expression of long intergenic non-protein coding RNA 467 (*linc00467*) is up-regulated by silenced N-MYC in NB cell line and its suppression promotes NB cell apoptosis [30].

*MYCN* amplification is a potent driver of NB oncogenesis. It encodes a transcription factor N-MYC, which first dimerizes to myc associated factor x (MAX) at E-box site of the promoter, and either activates or inhibits transcription of the target genes required in cell cycle, cell proliferation, neuronal differentiation, metabolism, and apoptosis in NB [31]. At the present time, lncRNAs that are altered by *MYCN* amplification and associated with prognostication for high-risk NB are largely unknown. Thus, here we performed differential expression study using microarray and RNA-seq based lncRNA and mRNA expression profiles between *MYCN* amplified and *MYCN* non-amplified subtypes in NB. The lncRNAs identified in both of the technologies, were further validated by quantitative reverse transcription polymerase chain reaction (RT-qPCR). Moreover, we performed co-expression study between lncRNAs and coding genes and identified lncRNA, *SNHG1* and coding gene, *TAF1D* to be one of the highly positively co-expressed pair. Furthermore, we conducted Kaplan-Meier survival analysis and univariate/multivariate statistical analysis by integrating the clinical information of NB patients to the expression value of *SNHG1*, and explored its clinical implications. Our results suggest that *SNHG1* can serve as a potential biomarker predicting NB patient's survival and an important resource for future functional characterization.

## RESULTS

### Transcriptome analysis between *MYCN* amplified and *MYCN* non-amplified NB

**Figure 1:**
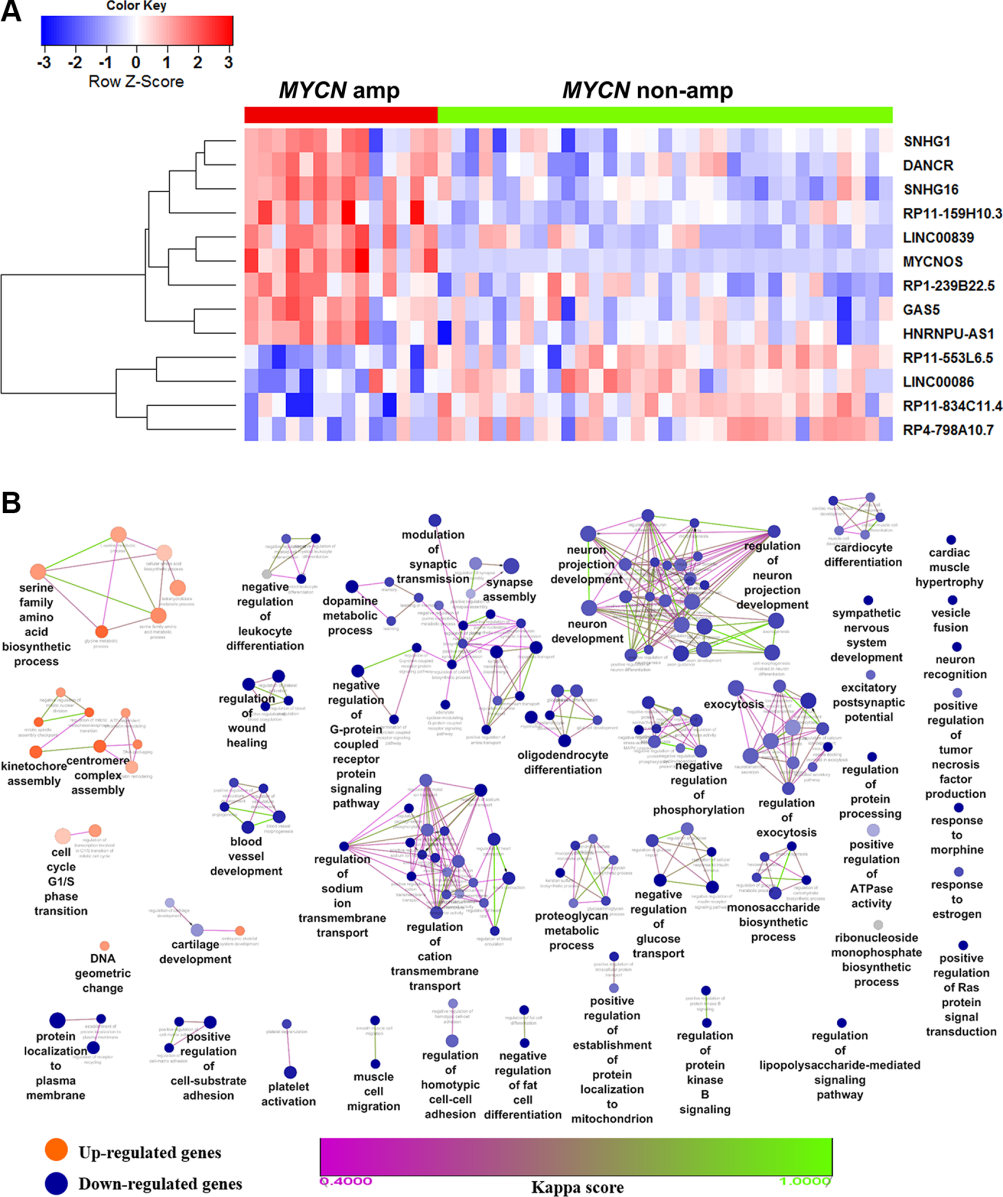
Heatmap of differentially expressed lncRNAs and biological functions of de-regulated coding genes in NB (**A**) Heatmap showing the expression values of lncRNAs found differentially expressed in *MYCN* amplified compared with *MYCN* non-amplified patient samples. Each column indicates a *MYCN* amplified (grouped in red bar) or non-amplified (green) patients samples. Each row represents the lncRNA ordered by hierarchical clustering analysis. Expression value of each lncRNA was scaled across samples and represented in a blue-red color scale. (**B**) Functional enrichment map of differentially expressed coding genes. Nodes represent GO terms and edges represent genes shared between GO terms (kappa score threshold = 0.4). Edge color gradient represents kappa score. Overview GO terms are shown by the highest statistically enriched term in each group formed by fusion of the GO terms sharing similar genes. Clusters with orange color are enriched functions for up-regulated genes while blue clusters are for down-regulated genes. Node color gradient represents the proportion of up (orange) or down-regulated (blue) genes associated with the term. A node (GO term) with equal proportion of up and down-regulated genes is represented in grey color.

To obtain the expression profile of lncRNAs, we first re-annotated the entire collection of probes for the Affymetrix HU133 plus 2.0 platform and identified 4,256 probes mapped to lncRNAs. Next, we carried out differential expression analysis of a microarray dataset (GEO accession GSE12460) with a total of 47 patients, out of which 14 were *MYCN* amplified and 33 were *MYCN* non-amplified (seventeen samples were removed with unknown *MYCN* status). We identified 21 lncRNAs and 591 coding genes to be differentially expressed (adjusted *P* ≤ 0.05 and fold change ≥ 2) in *MYCN* amplified samples compared with *MYCN* non-amplified ones in NB (Supplementary Table S1). Additionally, to enhance the data reliability, 13 lncRNAs (9 up-regulated and 4 down-regulated) that were reported in Gencode v.22 [18] were retained and shown as heatmap (Figure 1A). Next, to explore the biological functions of the differentially expressed coding genes, we performed a functional enrichment analysis. We found that biological processes such as serine family amino acid biosynthetic process, cell cycle, kinetochore assembly, centromere complex assembly, were significantly enriched for the up-regulated genes. These processes entail the involvement of genes in NB oncogenesis. In contrast, neuron projection development, neuron development, exocytosis, synapse assembly, biological processes got significantly enriched for the down-regulated genes. These processes entail their importance in the early stage of nervous system development (Figure 1B).

### Identification and validation of potential lncRNAs in NB

**Figure 2:**
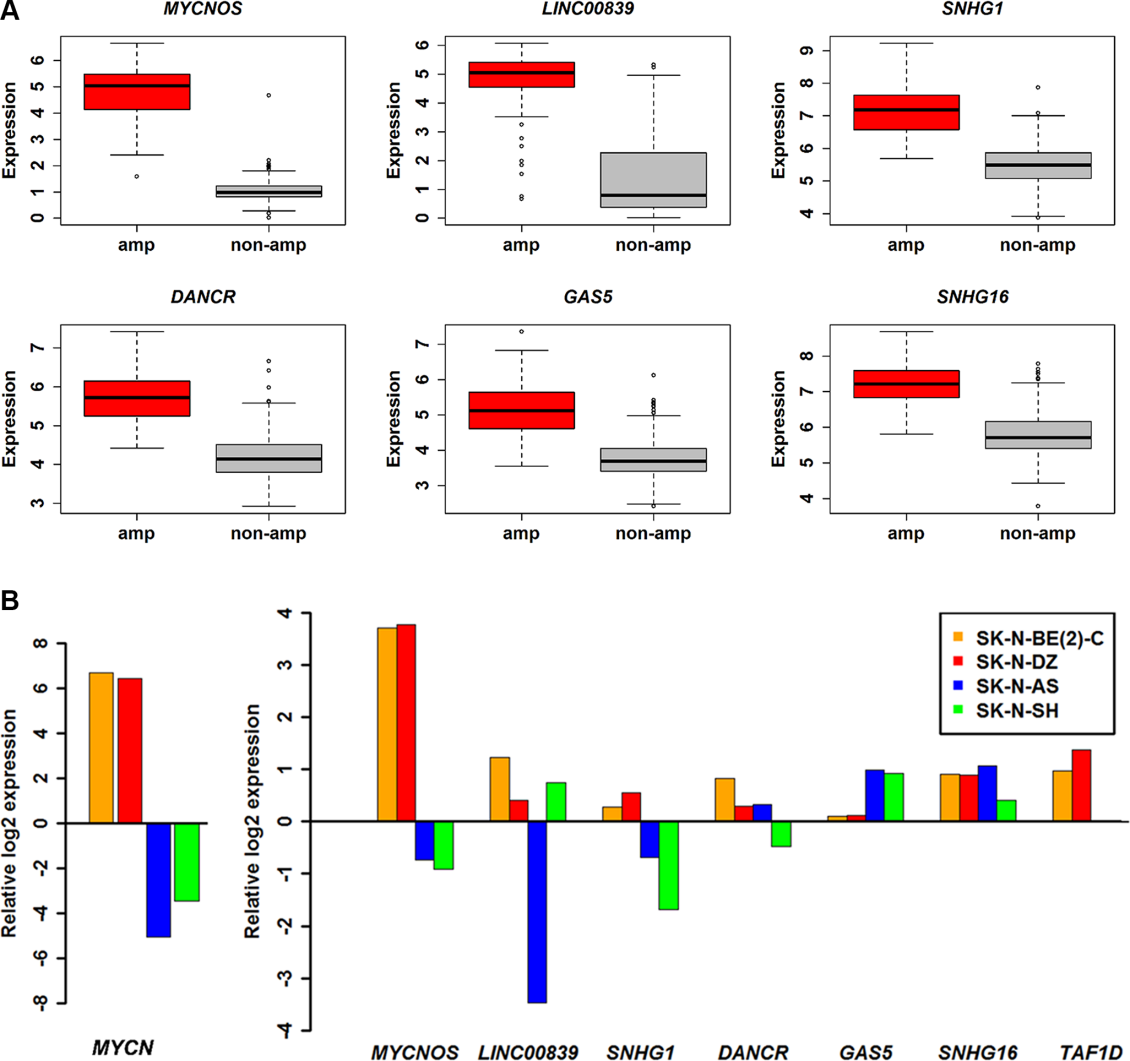
Expression profile of potential lncRNAs (**A**) Boxplot showing six lncRNAs expression profiles in *MYCN* amplified (*n* = 92) and *MYCN* non-amplified (*n* = 401). (**B**) Bar charts of expression levels measured by RT-qPCR in NB cell lines. The expression levels were normalized to endogenous *GAPDH* and relative to SK-N-F1 and displayed in log2 scale. *MYCN* and *TAF1D* are also shown for comparison.

Differential expression analysis of a RNA-sequencing (RNA-seq) dataset with GEO accession GSE62564, a total of 493 patients, out of which, 92 were *MYCN* amplified and 401 were *MYCN* non-amplified (five samples were removed with unknown *MYCN* status) was performed. We identified 90 lncRNAs and 1348 coding genes to be differentially expressed (adjusted *P* ≤ 0.05 and fold change ≥ 2) in the two subtype conditions (Supplementary Table S2). In the next step, fifty one differentially expressed lncRNAs were retained as they were also annotated in the Gencode v.22. Consistent with the previous studies RNA-seq has identified much more differentially expressed transcripts than microarray [32]. The transcripts shared between these two technologies are shown as a venn diagram (Supplementary Figure S1). Among the 51 lncRNAs from RNA-seq analysis, only 6 were identified in microarray analysis as well and termed as potential lncRNAs in NB (Table 1 and Figure 2A). We further used RT-qPCR to validate their differential expression in various NB cell lines such as *MYCN* amplified (SK-N-BE(2)-C, SK-N-DZ) and *MYCN* non-amplified (SK-N-AS, SK-N-F1, SK-N-SH). Interestingly, all the potential lncRNAs except *GAS5*, were up-regulated in *MYCN* amplified cell lines when compared with *MYCN* non-amplified cell line SK-N-F1 (Figure 2B). In the case of *GAS5*, the expression in *MYCN* amplified cell lines showed moderate up-regulation when compared with SK-N-F1. However, contradictory results were observed in SK-N-AS and SK-N-SH.

**Table 1:** Common potential lncRNAs identified by both microarray and RNA-seq differential expression analyses

lncRNA	RefSeq ID	Chromosome location	Microarray		RNA-seq	
			Fold change	*P* (corrected)	Fold change	*P* (corrected)
***MYCNOS***	NR_026766	Chr 2	5.55	8.09E-12	14.37	2.80E-231
***LINC00839***	NR_026827	Chr 10	4.67	3.42E-04	10.33	1.59E-73
***SNHG1***	NR_003098	Chr 11	2.47	2.05E-02	3.21	3.11E-81
***DANCR***	NR_024031	Chr 4	2.34	1.96E-03	2.91	3.11E-77
***GAS5***	NR_002578	Chr 1	2.43	2.30E-03	2.65	2.06E-66
***SNHG16***	NR_038110	Chr17	2.23	3.02E-04	2.64	2.77E-65

Abbreviation: *P* = *P*-value.

### Co-expression study between differentially expressed lncRNAs and coding genes in *MYCN* amplified and non-amplified NB

**Figure 3:**
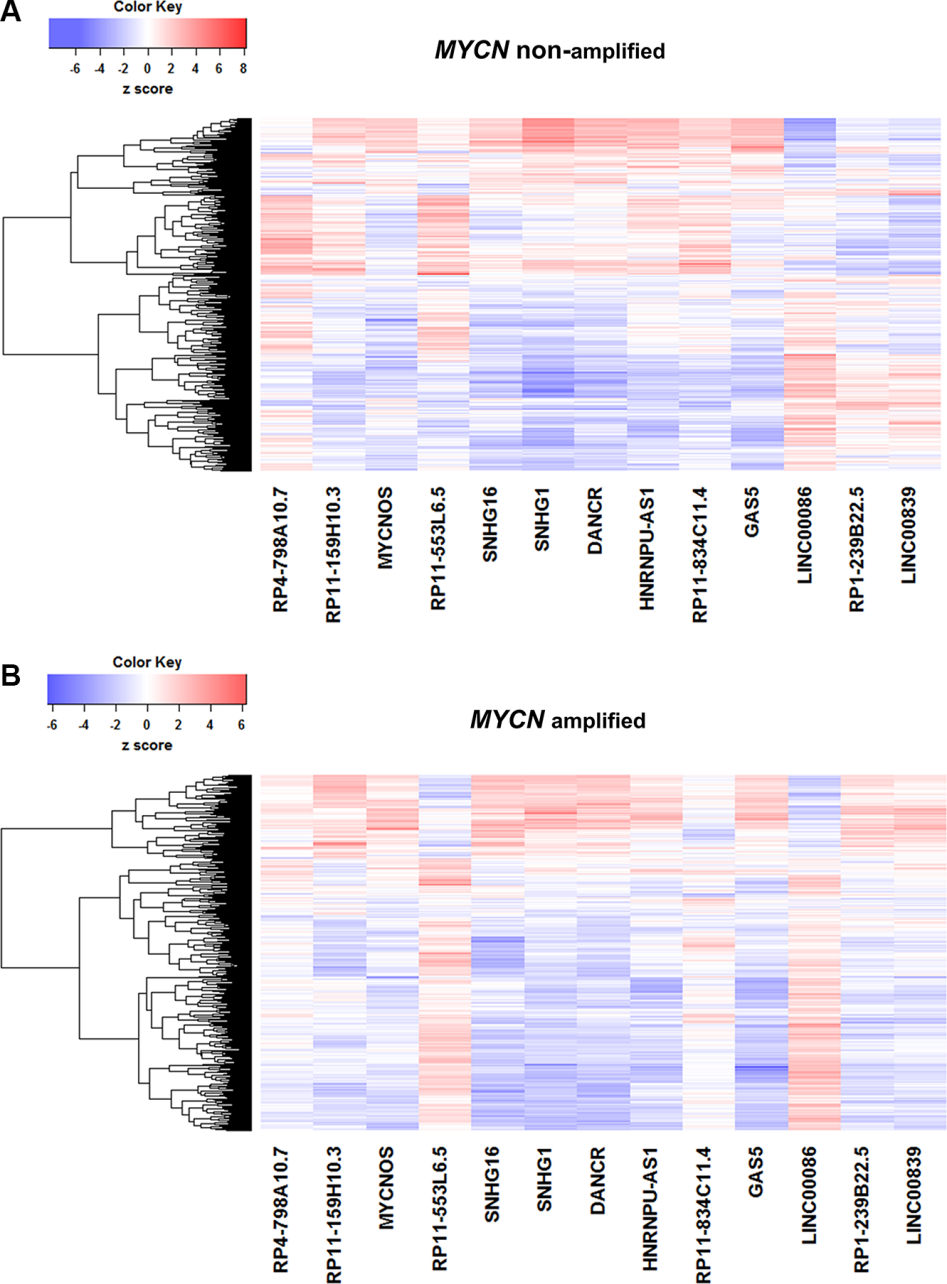
Correlation of lncRNA and coding gene expression profiles Fisher's *Z*-transformed score of Spearman's correlation coeffiecient (SCC) between lncRNAs (*n* = 13) and coding genes (*n* = 591) in (**A**) *MYCN* amplified and (**B**) *MYCN* non-amplified neuroblastoma tumor samples. Dendrograms of coding genes (rows) are displayed.

**Figure 4:**
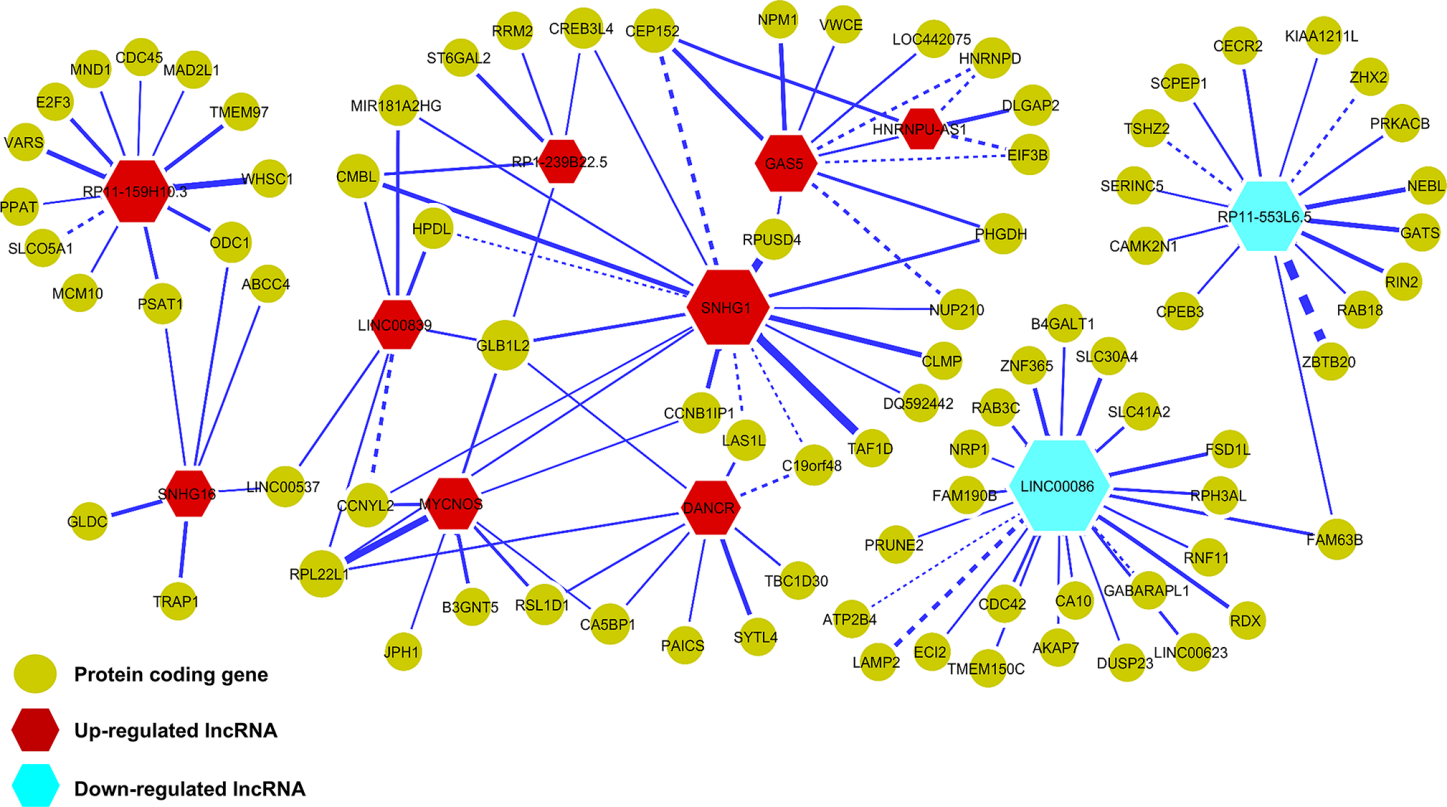
Co-expression network of lncRNAs and protein-coding genes in *MYCN* amplified neuroblastoma Nodes represent the lncRNAs and coding genes whereas edges represent the z-scores of expression correlation between lncRNAs and coding genes. Red nodes represent up-regulated lncRNAs and blue ones represent down-regulated lncRNAs. Yellow nodes represent protein-coding genes. Node size and edge width are proportional to the degree of a node and z-score, respectively. Dashed edges indicate that the connected lncRNA and coding gene pairs also appeared in the *MYCN* non-amplified network.

**Figure 5:**
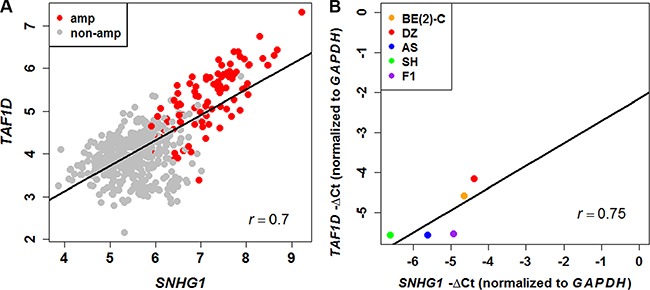
Positive correlation between *SNHG1* and *TAF1D* expression (**A**) Scatter plot of *SNHG1* and *TAF1D* expression levels in neuroblastoma patients measured by RNA-seq (*n* = 493) shows positive correlation. (**B**) The positive correlation was confirmed in NB cell lines via RT-qPCR (*r* = 0.75).

To decipher the transcriptional regulatory relationship, we calculated Spearman's correlation coefficient (SCC) between the expression values of microarray identified differentially expressed lncRNAs and coding genes in amplified and non-amplified conditions, respectively. Next, each correlation value was transformed into their z-score using Fisher's *Z*-transformation (Figure 3). Statistically significant co-expressed pairs were filtered out with z-score threshold ≥ 3.0 in both *MYCN* amplified and *MYCN* non-amplified conditions. We found 116 co-expression edges connecting 96 nodes (85 coding genes and 11 lncRNAs) in the *MYCN* amplified network (Figure 4) whereas 201 co-expression edges connecting 165 nodes (152 coding genes and 13 lncRNAs) in the *MYCN* non-amplified network (Supplementary Figure S2). In addition, to examine the chance of observing such amounts of connections in both networks, we randomized lncRNA and coding gene expression profiles by interchanging gene expression values within samples for 1,000 times, respectively. Then, we repeated the above co-expression analysis procedure to the randomized expression datasets for 10,000 times, and each time the same threshold cutoff was applied. The result depicted that random connections were less than the original ones with *P* < 0.0001 in both amplified and non-amplified cases (Supplementary Figure S3). Thus, the significant connections observed between lncRNA and coding genes in the original networks imply that there exist some underlying regulatory mechanisms which coordinate gene expression to execute a particular biological phenomena. Moreover, we filtered our original co-expression dataset, with SCC cut off threshold ≥ 0.8, and observed 39 co-expressed pairs. Among which, *SNHG1* (lncRNA) and *TAF1D* (coding gene) were found to be one of the highly positively correlated pairs with a significant z-score of 5.81 in a *MYCN* amplified subtype. We further tested this positive correlation in a RNA-seq dataset and also confirmed it by RT- qPCR (Figure 5). In consistence with the above findings, the correlation between *SNHG1* and *TAF1D* was also significant when it was evaluated by mutual rank (MR) in the four NB datasets with GEO accession, GSE13136 (*n* = 30), GSE12460 (*n* = 47), GSE16476 (*n* = 88) and GSE62564 (*n* = 493) (Table 2).

**Table 2:** Correlation and mutual rank between *SNHG1* and *TAF1D* in various microarray and RNA-seq datasets

Datasets	SCC (*MYCN* amp)	SCC (*MYCN* non-amp)	MR (*MYCN* amp)	MR (*MYCN* non-amp)
**GSE12460 (*n* = 47) *Microarray***	0.95	0.38	1	846
**GSE16476 (*n* = 88) *Microarray***	0.81	0.46	12	110
**GSE62564 (*n* = 493) *RNA-seq***	0.75	0.23	2	1087
**GSE13136 (*n* = 30) *Microarray***	0.6	0.65	927	94

Abbreviations: SCC = Spearman's correlation coefficient, MR = mutual rank.

### *SNHG1* is up-regulated in high-risk NB

**Figure 6:**
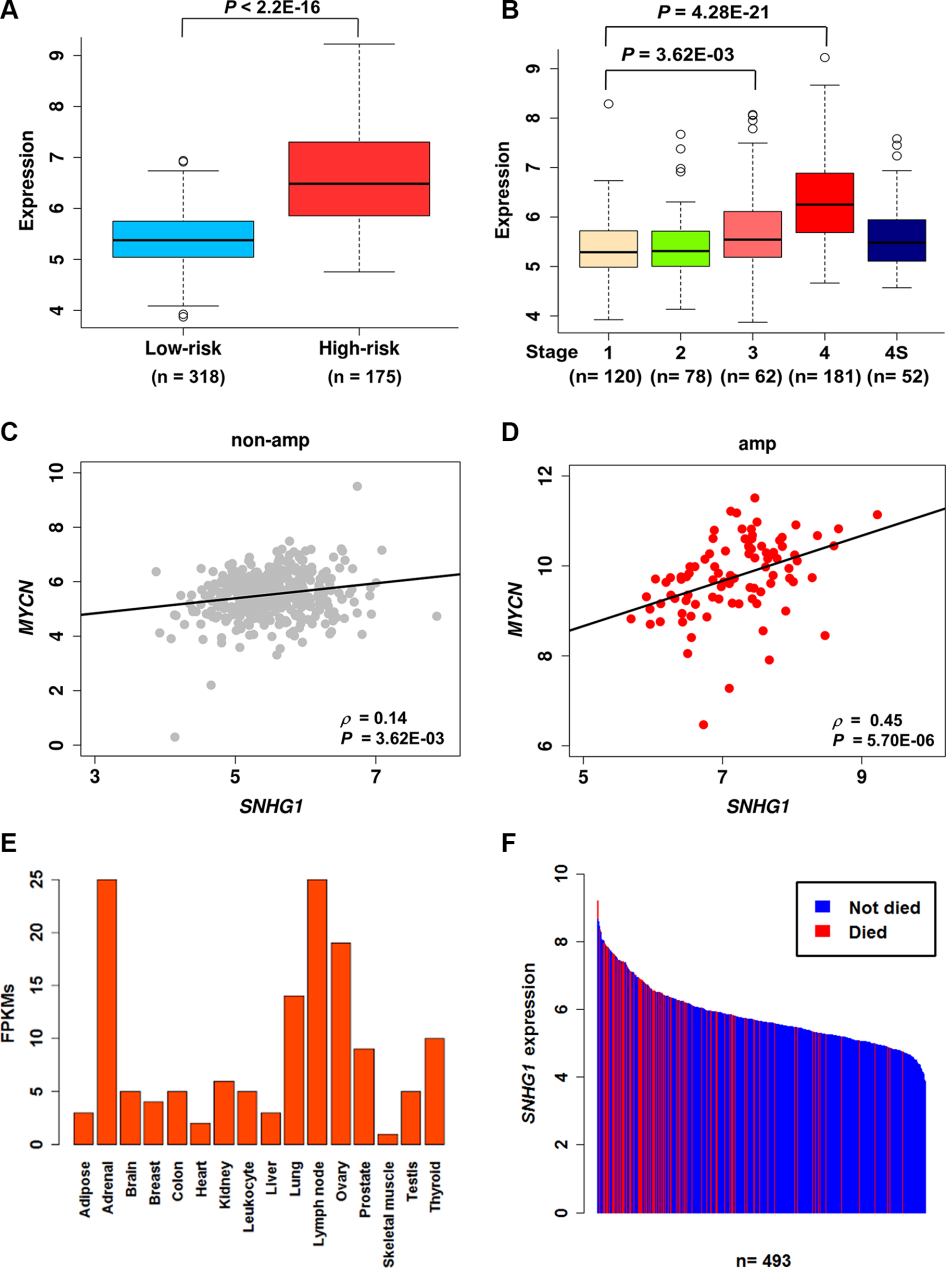
*SNHG1* is a prognostic marker for neuroblastoma (**A**, **B**) Boxplots showing the normalized log2RPM expression values of *SNHG1* in different risk groups and stages in a NB cohort (*n* = 493). The *P*-values presented were determined by Mann-Whitney-Wilcoxon test (A) and Dunn's multiple comparison test (B). (**C**, **D**) Scatter plots showing the correlation between *MYCN* and *SNHG1* in *MYCN* non-amplified (*n* = 401) and *MYCN* amplified patients (*n* = 92). SCC and the corresponding *P*-values are displayed. (**E**) Bar chart showing the ordered expression levels across 16 normal human tissues, based on the RNA-seq data from the Illumina Body Map project. (**F**) Bar chart showing the ordered expression levels of *SNHG1* per survival status of the patient. Here, blue and red bars represent patients did not die and died of disease, respectively.

*SNHG1* and its co-expressed coding gene, *TAF1D* was also found to be significantly up-regulated in the high-risk NB patients (Figure 6A, Supplementary Figure S4A). We next investigated their expression levels in different stages of NB based on the International Neuroblastoma Staging System (INSS) and discovered that, as compared to stages 1–3, *SNHG1* showed a significant higher expression in stage 4. In addition, compared to stage 4S, which is also a metastatic stage found in children younger than one year, stage 4 still had significantly higher *SNHG1* expression (*P* = 3.93E-07) (Figure 6B, Supplementary Figure S4B). This implies that highly expressed *SNHG1* might play a role in the progression of NB. Moreover, the expression of *SNHG1* and *MYCN* was found to be positively correlated in both amplified and non-amplified subtype conditions, which were further validated by RT-qPCR (Figures 2B, 6C–6D, Supplementary Figure S4C–S4D). This suggests that *SNHG1* is not only correlated with *MYCN* amplification but also with *MYCN* expression. Moreover, our ChIP-seq data analysis observed MYCN binding sites in the promoter of *SNHG1* (Supplementary Figure S5) [33]. Furthermore, previous study has pointed out that lncRNAs possess tissue-specific expression [34]. We examined the RNA-seq expression of *SNHG1* across 16 normal human tissues obtained from Illumina Human BodyMap 2.0 project, and found that it also shows tissue-specific expression predominantly in the adrenal gland, a common site for origin of NB (Figure 6E). To address the possibility that expression of *SNHG1* might play a role in the patient survival status, we have shown a bar plot of the ordered expression of *SNHG1* per survival status of the NB cohort (*n* = 493) (Figure 6F). This indicates that high expression of *SNHG1* is associated with the disease outcome.

### High expression of *SNHG1* is critical to patient survival

**Figure 7:**
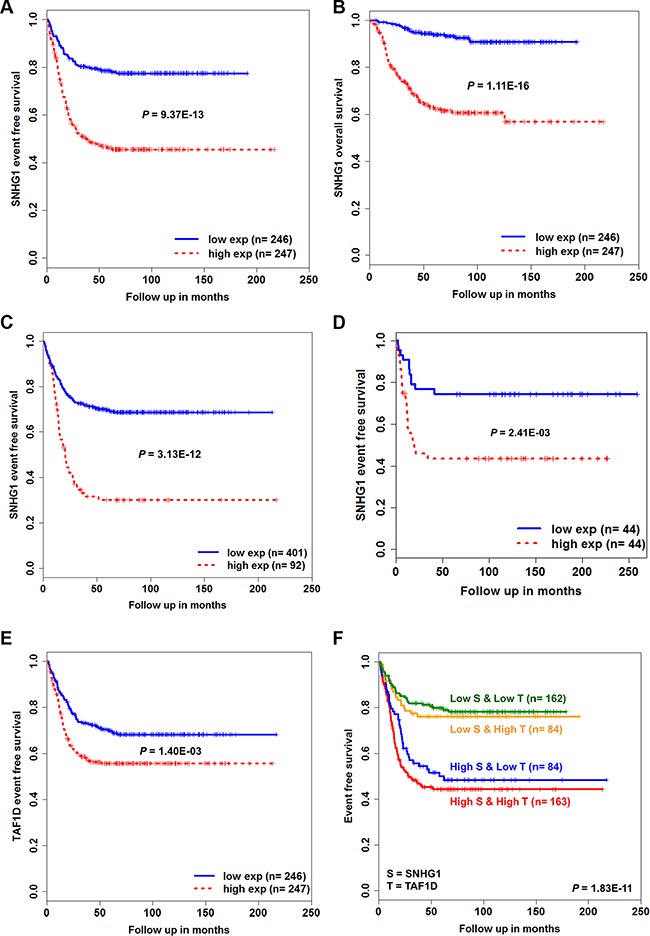
Kaplan-Meier survival analysis for NB patients The Kaplan-Meier plots for (**A**) event-free survival (EFS) and (**B**) overall survival (OS) of low-expression versus high-expression groups based on the median *SNHG1* expression level of GSE62564 patients (*n* = 493). (**C**) Kaplan-Meier curves of low-expression (*n* = 401) versus high-expression group (*n* = 92) based on the ordered expression of *SNHG1*. (**D**) Kaplan-Meier plot curve for EFS of GSE16476 patients (*n* = 88) (**E**) Kaplan-Meier plot for EFS of low-expression versus high-expression group based on the median expression of *TAF1D* (*n* = 493). (**F**) Kaplan-Meier plot for EFS of combinatorial low and high expression of both *SNHG1* and *TAF1D* (*n* = 493). The *P*-values were obtained by log-rank (Mantel-Haenszel) test.

To understand the prognostic value of *SNHG1*, we performed a Kaplan-Meier survival analysis on the expression value of *SNHG1* (*n* = 493). First, we classified patients into low-expression (*n* = 246) and high-expression (*n* = 247) groups based on the median expression of *SNHG1*. We observed that patients in the high-expression group displayed poorer event-free survival (EFS) (*P* = 9.37E-13) and overall survival (OS) (*P* = 1.11E-16) than those in the low-expression group (Figure 7A–7B). Second, we ordered *SNHG1* expression and randomly selected the top 92 expression values and classified them into a high-expression group, whereas the remaining 401 into a low-expression group. This arrangement is irrespective of the patient's *MYCN* or risk status. The high-expression leads to significantly poorer EFS than low expression (*P* = 3.13E-12) (Figure 7C). We observed consistency in our results based on the analysis performed in an independent cohort (*n* = 88) (Figure 7D, Supplementary Figure S6A). Importantly, the multivariate Cox regression analysis revealed that high expression of *SNHG1* can act as an independent prognostic biomarker predicting EFS in NB (*n* = 493, hazard ratio = 1.58, *P* = 2.36E-02) (Table 3).

**Table 3:** Cox regression analysis of *SNHG1* with established risk factors in GSE62564 NB cohort (*n*= 493)

Variables	EFS			OS		
	HR	95% CI	*P*	HR	95% CI	*P*
*Univariate model*						
***MYCN (amp vs non-amp)***	3.22	(2.35–4.41)	3.19E-13	7.80	(5.27–11.55)	< 2e-16
***Stage (4 vs 1,2,3 & 4S)***	3.88	(2.86–5.27)	< 2e-16	8.56	(5.38–13.63)	< 2e-16
***Age (≥ 18 months vs < 18 months)***	3.33	(2.46–4.52)	9.88E-15	8.55	(5.24–13.94)	< 2e-16
***Age (cont.)***	1.00	(1–1)	5.32E-13	1.00	(1–1)	3.22E-15
***Sex (male vs female)***	0.90	(0.69–1.19)	4.69E-01	0.94	(0.65–1.36)	7.46E-01
***SNHG1 (≥ 5.65 vs < 5.65)***	3.03	(2.2–4.18)	1.09E-11	6.54	(3.93–10.89)	5.25E-13
***SNHG1 (cont.)***	1.76	(1.53–2.02)	1.78E-15	2.37	(1.99–2.81)	< 2e-16
***TAF1D (≥ 4.11 vs < 4.11)***	1.62	(1.2–2.17)	1.55E-03	1.89	(1.27–2.8)	1.69E-03
***TAF1D (cont.)***	1.58	(1.32–1.89)	5.11E-07	2.10	(1.69–2.62)	2.50E-11

*Multivariate model*						
***MYCN (amp vs non-amp)***	1.34	(0.9–2.01)	1.51E-01	2.80	(1.65–4.75)	1.34E-04
***SNHG1 (≥ 5.65 vs < 5.65)***	1.58	(1.06–2.35)	**2.36E-02**	2.22	(1.21–4.07)	**1.02E-02**
***TAF1D (≥ 4.11 vs < 4.11)***	1.24	(0.87–1.77)	2.36E-01	0.93	(0.56–1.54)	7.67E-01
***Stage (4 vs 1,2,3 & 4S)***	2.10	(1.43–3.09)	1.68E-04	2.63	(1.51–4.56)	6.05E-04
***Age (≥ 18 months vs < 18 months)***	1.97	(1.39–2.8)	1.60E-04	3.54	(2.04–6.12)	6.60E-06

***MYCN (amp vs non-amp)***	1.85	(1.08–3.17)	2.57E-02	5.19	(2.72–9.93)	6.34E-07
***SNHG1 (cont.)***	1.72	(1.33–2.24)	**4.35E-05**	1.95	(1.38–2.77)	**1.68E-04**
***TAF1D (cont.)***	0.76	(0.58–0.99)	4.15E-02	0.59	(0.43–0.83)	1.89E-03

Abbreviations: EFS = event-free survival; OS = overall survival; HR = hazard ratio; CI = confidence interval; *P* = *P*-value; cont. = continuous expression.

a In both univariate and multivariate Cox regression analyses, the gene median expression cutoff was used to divide patients into high and low expression groups.

b *P*-value in bold typeface indicates statistical significance (*P* < 0.05).

Further, to examine the prognostic value of *SNHG1* co-expressed coding gene *TAF1D*, we again classified patients into low-expression (*n* = 246) and high-expression groups (*n* = 247) based on the median expression of *TAF1D*. We found that high-expression of *TAF1D* also leads to poor patient EFS (*P* = 1.40E-03) (Figure 7E, Supplementary Figure S6B).

### Combinatorial expression of *SNHG1* and *TAF1D* affects prognosis

We next sought to understand whether co-expressed *SNHG1* and *TAF1D* have a combinatorial effect on the patient survival outcome. To achieve this, all patients were divided into four groups based on high or low expression status of *SNHG1* and *TAF1D* in each patient: Group 1 (high-expression of both *SNHG1* and *TAF1D*, *n* = 163), Group 2 (high-expression of *SNHG1* and low-expression of *TAF1D*, *n* = 84), Group 3 (low-expression of *SNHG1* and high- expression of *TAF1D*, *n* = 84) and Group 4 (low-expression of both *SNHG1* and *TAF1D*, *n* = 162). We found EFS in the four groups as 44%, 48%, 76% and 78% at end points, respectively (Figure 7F). In addition, the interaction between the expression values of *SNHG1* and *TAFID* was significant in the multivariate Cox analysis (Table 4). We also evaluated our hypothesis by creating a risk-score formula, which is a linear combination of gene expression values weighted by their univariate Cox regression coefficient for EFS as follows: Risk Score = (0.5648381 * expression of *SNHG1*) + (0.459009 * expression of *TAF1D*) for each patient. Next, median risk-score was used as a cutoff to divide patients into low-risk (*n* = 246) and high-risk group (*n* = 247). We observed that patients in the high-risk group is significantly associated with poor patient survival (*P* = 4.70E-10) (Supplementary Figure S7). Interestingly, this interaction effect was found to be independent when assessed in the multivariate Cox analysis (Table 4 and Supplementary Table S3). We further asked whether the hazard rate obtained is constant over time. We performed tests of proportional hazard for each variable and found no violation of the assumption (Table 5 and Supplementary Table S4). Collectively, these results indicate that highly expressed *SNHG1* has a dominant influence on the patient prognostication and upon its interaction with *TAF1D* might worsen patient survival outcome.

**Table 4:** Multivariate Cox-regression analysis for combinatorial effect of *SNHG1* & *TAF1D* with established factors in GSE62564 NB cohort

Variables	EFS		
	HR	95% CI of HR	*P*
***MYCN (amp vs non-amp)***	1.26	(0.76–2.12)	3.71E-01
**Stage *(4 vs 1,2,3 & 4S)***	2.16	(1.46–3.2)	1.25E-04
**Age *(≥ 18 months vs < 18 months)***	2.00	(1.4–2.85)	1.28E-04
***SNHG1 (cont.)***	3.40	(1.39–8.33)	7.51E-03
***TAF1D (cont.)***	4.71	(1.36–16.34)	1.47E-02
SNHG1 (cont.) * TAF1D (cont.)	0.80	(0.67–0.96)	**1.54E-02**

***MYCN (amp vs non-amp)***	1.36	(0.93–1.97)	1.10E-01
**Stage *(4 vs 1,2,3 & 4S)***	2.23	(1.56–3.2)	1.22E-05
**Age *(≥ 18 months vs < 18 months)***	1.97	(1.39–2.81)	1.67E-04
***SNHG1 & TAF1D risk score (≥ 5.02 vs < 5.02)***	1.76	(1.22–2.52)	**2.25E-03**

a *P*-value in bold typeface indicates statistical significance (*P* < 0.05)

**Table 5:** Cox proportional hazard analysis of EFS in NB patients

Variable	rho	chisq	*P*
***MYCN (amp vs non-amp)***	0.12	2.64	1.04E-01
***SNHG1 (≥ 5.65 vs < 5.65)***	0.07	0.82	3.66E-01
***TAF1D (≥ 4.11 vs < 4.11)***	−0.12	2.60	1.07E-01
**GLOBAL**	NA	5.46	1.41E-01

a The non-significant *P*-value for each variable and the global test as a whole indicates that the hazard ratio obtained is constant over time.

## DISCUSSION

Recently, lncRNAs due to their abundance and functions have gained lots of scientific interests. They are pervasively transcribed and classified into a diverse range of transcripts based on their orientation to neighboring protein-coding genes as antisense, intergenic, overlapping, intronic or processed [18]. Abruptly expressed lncRNAs regulate their target gene by acting in *cis* or in *trans* manner [25, 35–38]. Accumulating evidence has linked dysregulated lncRNAs functionally either as oncogenes or tumor suppressors in various cancer progression and development [39]. However, only few reports have discussed their contribution in NB. *MYCN* oncogene amplification, is still one of the most powerful predictors for the fatal outcome in NB. It exhibits oncogenic activity by altering the expression of its target genes. To date, very little is known about lncRNAs that are altered by *MYCN* amplification and associated with patient prognostication, which along with *MYCN* can augment NB pathogenesis. We hypothesized that genes which are significantly differentially expressed are directly or indirectly regulated by *MYCN* amplification. Thus, we performed differential expression analysis between *MYCN* amplified and *MYCN* non-amplified NB. The heatmap of lncRNA expression profile generated by hierarchical clustering showed a clear separation of *MYCN* amplified samples from the *MYCN* non-amplified ones.

In contrast to microarray, RNA-seq extensively detect the gene abundance with higher sensitivity. Therefore, we next screened lncRNAs and protein-coding genes based on the RNA-seq differential expression analysis. We identified common transcripts detected by both platforms. Employing this approach, we discovered a potential dysregulated lncRNA set in NB, comprising of six lncRNAs followed by successful RT-qPCR validation of each lncRNA except *GAS5*. Here, *MYCNOS* and *SNHG1* showed highly positive correlation with *MYCN* expression.

The lncRNA, mycn opposite strand (*MYCNOS*) physically interacts with a transcription factor CCTF and epigenetically enhances *MYCN* expression resulting in loss of differentiation, tumor progression and invasion in NB cells [40]. Small nucleolar RNA host gene 16 (*SNHG16*) also known as non-coding RNA expressed in aggressive neuroblastoma (*ncRAN*), is located in chromosome 17q and its high expression is associated with poor outcome of NB patient [41]. Differentiation antagonizing non-protein coding RNA (*DANCR*) possess stem-cell like properties and critical to patient survival of hepatocellular carcinoma (HCC). Over-expression of *DANCR* enhances stemness features and tumorigenesis in HCC cells [42]. Small nucleolar RNA host gene 1 (*SNHG1*) is up-regulated in lung cancer, breast cancer and HCC [43–45]. High expression of *SNHG1* is associated with poor prognosis of HCC patients [45]. However, the functional characterization and clinical implication of *LINC00839*, *DANCR* and *GAS5* in NB are still unknown. Future research on these candidate lncRNAs will promote better understanding of NB etiology.

Typically lncRNAs are highly correlated with their neighboring protein-coding genes [34]. Studies have pointed out that regulatory mechanism such as copy number variation, DNA methylation and transcription factors can induce the lncRNAs and protein-coding genes to be co-expressed together and amplify the cancer pathogenesis [46]. Therefore we applied co-expression analysis study on lncRNAs and coding genes in NB. To identify statistically significant co-expressed pairs in both amplified and non-amplified conditions, we converted each correlation value into z-score. This procedure has also been applied in previous studies [47]. By applying a significant threshold we identified *SNHG1* and *TAF1D* as one of the highly co-expressed pairs which were further validated by RT-qPCR.

*SNHG1* is located in 11q12.3 region of the chromosome whose deletion is linked with poor prognosis in high risk NB tumors [48]. To investigate the prognostic impact of *SNHG1*, we performed survival analysis by integrating *SNHG1* expression and clinical outcome of NB patients in the RNA-seq datasets. A clear separation was observed in the survival curves between patients who were divided based on either *SNHG1* median expression cutoff or upon top 92 and remaining 401 ordered expression values. In addition, the prognostic value was also reproduced in an independent cohort of NB patients in microarray datasets. Moreover, our multivariate Cox-regression result revealed that, by incorporating established clinical markers such as *MYCN* amplification, stage, and age into analysis, *SNHG1* high expression still displayed a significant poor hazard rate for both overall and event-free survivals. Collectively these data indicated that highly expressed *SNHG1* can act as a prognostic biomarker in predicting clinical outcome of NB patients. Additionally, we also showed that correlated *SNHG1* and *TAF1D* expression values might have an interaction effect on the patient survival outcome, which was revealed by significant *P*-values of their interaction and risk-score in the multivariate Cox analysis. However, this effect needs to be further validated by experiments. Using risk score model to investigate multiple gene effect on patient outcome has also been applied by several studies [49, 50].

*SNHG1* is a host to 8 small nucleolar RNAs (snoRNAs), namely *SNORD22* and *SNORD25-31*, residing within its introns [51]. Studies have shown emergence of snoRNAs as potential regulators in cancer development [52]. Therefore, it is important to evaluate whether the prognostic value and prospective biological functions of *SNHG1* are independent of snoRNAs. Here, we examined their expression levels in the RNA-seq data and found the snoRNA expression to be substantially low compared with their host gene (Supplementary Figure S8). However, this hypothesis needs further experimental support. In addition, these snoRNAs did not pass our cutoff threshold for differential expression and thus were filtered out from the analysis.

Taken together, our integrative analysis reveals that *SNHG1* could be a prognostic biomarker that independently predicts poor clinical outcome for EFS in NB patients. Additionally, *SNHG1* is driven by N-MYC and regulated by *MYCN* amplification. *SNHG1* expression is positively correlated with *MYCN* expression in both *MYCN* amplified and *MYCN* non-amplified NB tumors. These empirical evidences open up new research opportunities to elucidate the functional characterization of this novel lncRNA marker, which will improve our knowledge in deeper understanding of its functional role in the NB etiology and its potential as therapeutic target for NB intervention.

## MATERIALS AND METHODS

### Preprocessing of the microarray dataset and detection of differentially expressed lncRNAs and coding genes

The raw CEL files were downloaded from the NCBI GEO database. The samples with unknown *MYCN* status were removed. The normalized and log2 transformed expression value of probes were extracted using Robust Multi-array Average (RMA) normalization algorithm. Next, the limma R package [53] was applied to identify differentially expressed coding genes and lncRNAs in *MYCN* amplified compared with *MYCN* non-amplified subtype conditions. For the case of multiple probes representing the same gene, their averaged expression were taken for further analyses.

### Functional enrichment analysis

ClueGO [54], a Cytoscape plug-in was used for the interpretation of functions enriched for the differential coding genes. Statistical parameters such as right-sided hypergeometric test, *P* < 0.05 with Benjamini-Hochberg correction, GO levels between 6 to 14 and kappa score threshold of 0.4, were applied to identify the gene ontology (GO) terms enriched for the up-regulated and down-regulated genes.

### Identification of differentially expressed lncRNAs and coding genes in the RNA-seq dataset

The log2RPM normalized RNA-seq dataset and patients clinical data with GEO accession GSE62564 were downloaded. The samples with unknown *MYCN* status were removed. To avoid negative log2 expression values, the intensities were converted back to their original raw expression and increased with one and then log2 transformed. The coding-gene and lncRNA expression data were extracted based on RefSeq ID annotations, which identified 34,255 and 6,260 coding genes and lncRNAs transcripts. Next, limma package with the same threshold as described earlier was applied for the detection of differentially expressed transcripts. For the case of gene with transcript variant, high standard deviation transcipt were taken for further analyses.

### Co-expression analysis and Fisher's Z-transformation

We calculated Spearman's correlation coefficients (SCC) between the expression values of differentially expressed coding genes and lncRNAs. Next, Fisher transformation was applied to each of the calculated correlation as follows,

$$F(r)=\frac{1}{2}log\left(\frac{1+r}{1-r}\right)$$

where *F*(*r*) is the Fisher transformed score of *r*, the SCC of lncRNA and coding gene pair. Further, z-score of the Fisher transformed SCC is calculated by the following expression,

$$Z=\sqrt{\frac{N-3}{1.06}}F\left(r\right)$$

where *Z* is the z-score of *r* and *N* is the sample size. Larger z-score implies the correlation is statistically significant. The threshold for z-score was set to ≥ 3.0 and co-expression network was constructed in Cytoscape 3.3.0.

### Mutual rank calculation

Mutual rank (MR) is a measure of the geometric average between the correlation rank of gene A to gene B and gene B to gene A [55]. Here, the *MYCN* amplified and *MYCN* non-amplified samples were separated for each dataset. Next, SCC values were calculated between all the lncRNAs and coding genes present in each dataset. Further, the absolute highest correlation was ranked with smallest rank values. The MR was calculated by the following expression,

$$\text{MR}(\text{AB})=\sqrt{\text{Rank}\left(A\to B\right)\in \text{Rank}\left(B\to A\right)}$$

### Cell line culture

Human neuroblastoma cell lines SK-N-DZ, SK-N-SH, and SK-N-BE(2)-C were purchased from American Type Tissue Collection (ATCC). SK-N-AS and SK-N-F1 neuroblastoma cell lines were kindly provided by Dr. Yung-Feng Liao (Institute of Cellular and Organismic Biology, Academia Sinica). All cell lines were cultured in Dulbecco's Modified Eagle's Medium (DMEM) supplemented with 10% fetal bovine serum (FBS) (Biological Industries). Cells were grown at 37°C under 5% CO_2_ atmosphere.

### Cellular RNA extraction and reverse transcription

Neuroblastoma cells were homogenized in TRIzol reagent (Invitrogen) and cellular RNA was extracted using Direct-zol^TM^ RNA MiniPrep kit (Zymo Research) following the manufacturer's protocol. RNA concentration was determined by NanoDrop ND-1000 (NanoDrop Technologies) and RNA quality was checked by 1% agarose gel electrophoresis. One microgram total RNA of each sample along with oligo(dT)_18_ and random hexamer primer was reverse transcribed to cDNA in a final volume of 20 μl using RevertAid^TM^ H Minus First Strand cDNA Synthesis Kit (Thermo Scientific). The synthesized cDNA was stored in −80°C until use.

### Real-time quantitative PCR

The cDNA of each cell line was amplified using iQ^TM^ SYBR Green Supermix (Bio-rad) and CFX96 Real-Time PCR System (Bio-rad). Each lncRNA in each cell line was repeated in triplicates. The average expression (ΔCt) of each lncRNA was normalized to average *GAPDH* expression. The relative expression (ΔΔCt) was calibrated to ΔCt of SK-N-F1. The primer sequence for each lncRNA used in the study is listed in the Supplementary Table S5.

### Survival analysis

The Kaplan-Meier event-free survival (EFS) and overall survival (OS) analysis was performed for the two groups of patients, classified on the basis of median gene expression value. The significance of the survival curve was assessed using the log-rank (Mantel-Haenszel) test. Next, the risk association of the lncRNA expression among several known risk factors was determined using univariate and multivariate Cox regression analyses. Proportional hazard assumption for the variable was computed by cox.zph function embedded in the R survival package.

## SUPPLEMENTARY MATERIALS FIGURES AND TABLES








