# Supplementary material for: Co-expression analysis identifies long noncoding RNA SNHG1 as a novel predictor for event-free survival in neuroblastoma
Source: Oncotarget. 2016 Aug 9;7(36):58022–37. doi: 10.18632/oncotarget.11158 (PMC5295409; doi:10.18632/oncotarget.11158)
Supplement: Supplementary file 1 [file oncotarget-07-58022-s001.pdf]

Co-expression analysis identifies long noncoding RNA *SNHG1* as a novel predictor for event-free survival in neuroblastoma

Supplementary Materials

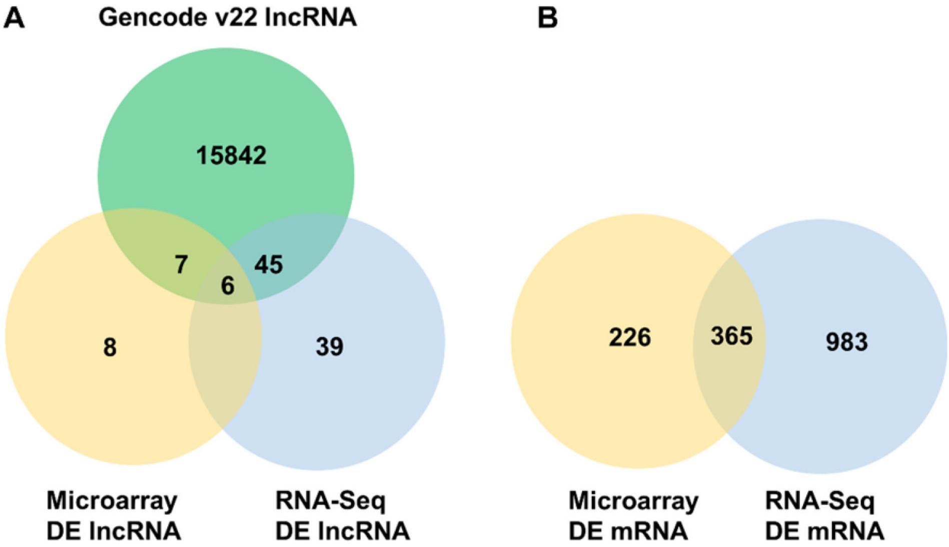

Supplementary Figure S1: Venn diagram showing (A) The number of differentially expressed lncRNAs shared between Gencode v.22 lncRNAs and microarray as well as RNA-Seq differential expression analyses (B) The number of differentially expressed protein-coding genes shared between microarray and RNA-Seq differential expression analyses.

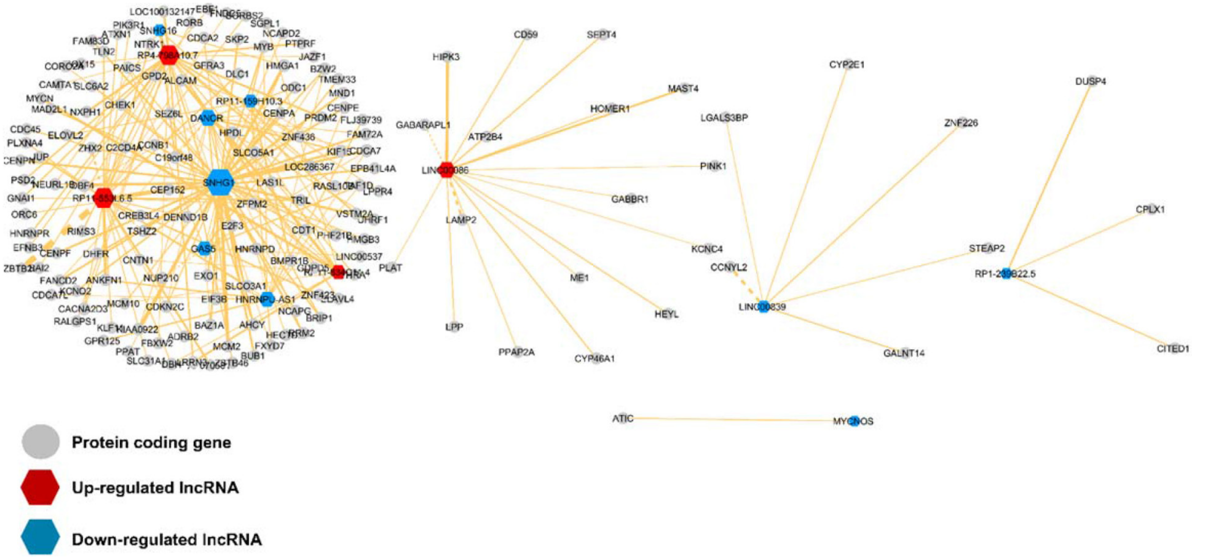

Supplementary Figure S2: Co-expression network of lncRNAs and protein-coding genes in the *MYCN* non-amplified neuroblastoma. Nodes represent lncRNAs and coding genes whereas edges represent z-scores of expression correlation between lncRNAs and coding genes. Red nodes represent up-regulated lncRNAs and blue ones represent down-regulated lncRNAs. Grey nodes represent protein-coding genes. Node size and edge width are proportional to degree of a node and z-score respectively. Dashed edges indicate that the connected lncRNA and coding gene pairs also appeared in the *MYCN* amplified network.

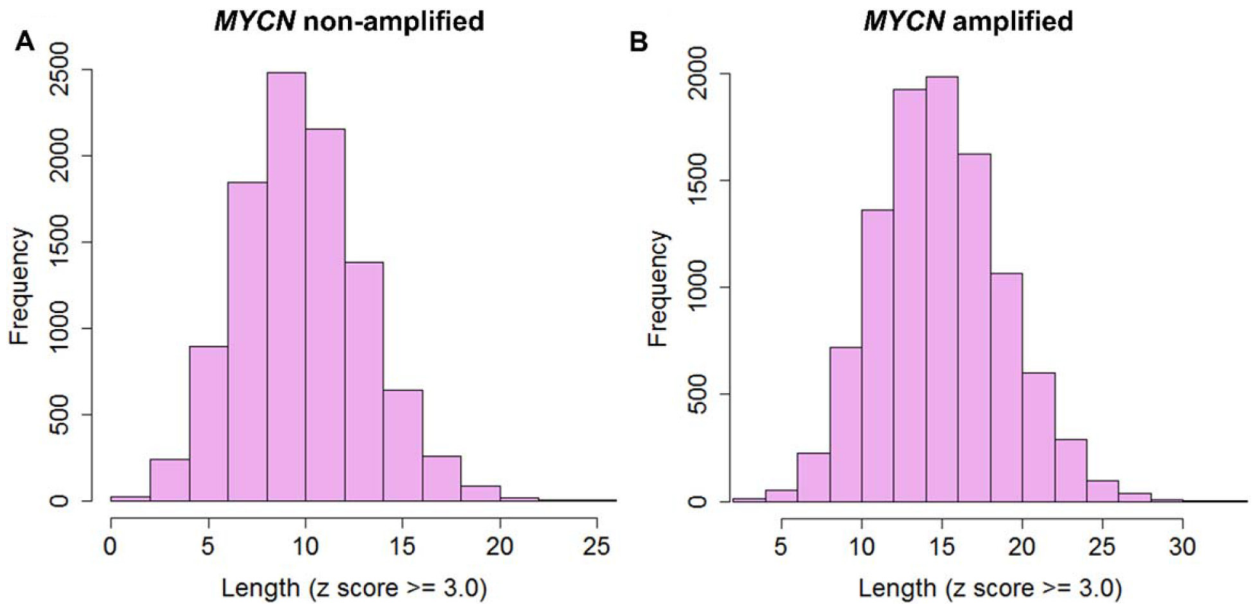

Supplementary Figure S3: (**A**, **B**) Histogram showing the frequency of connections observed between randomized co-expressed lncRNAs and coding genes in *MYCN* non-amplified and *MYCN* amplified conditions.

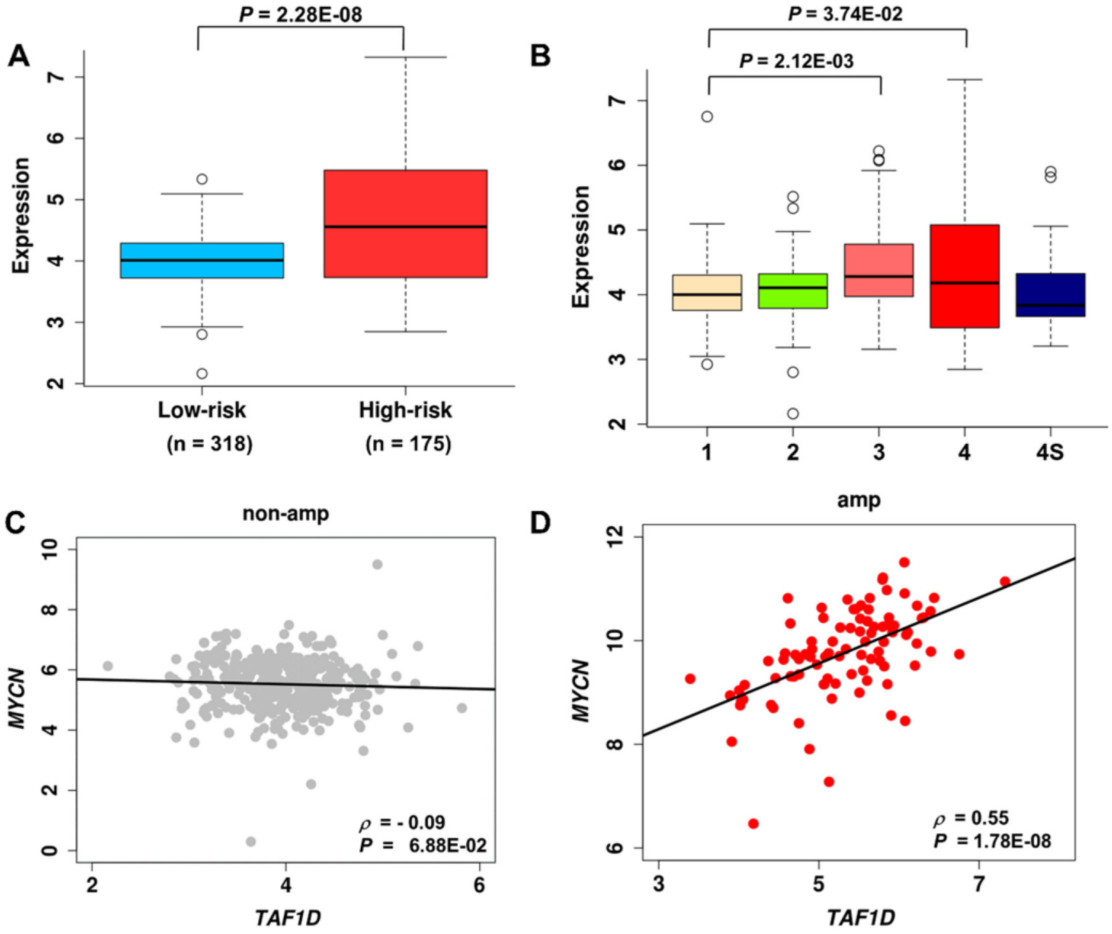

Supplementary Figure S4: (**A**, **B**) Boxplots showing the normalized log2RPM expression values of *TAF1D* in different risk groups and stages in neuroblastoma cohort ($n = 493$). The *P*-values presented were determined by Mann-Whitney-Wilcoxon test (A) and Dunn's multiple comparison test (B). (**C**, **D**) Scatter plot showing the correlation between *MYCN* and *TAF1D* in *MYCN* non-amplified ($n = 401$) and *MYCN* amplified ($n = 92$). SCC and the corresponding *P*-values are displayed.

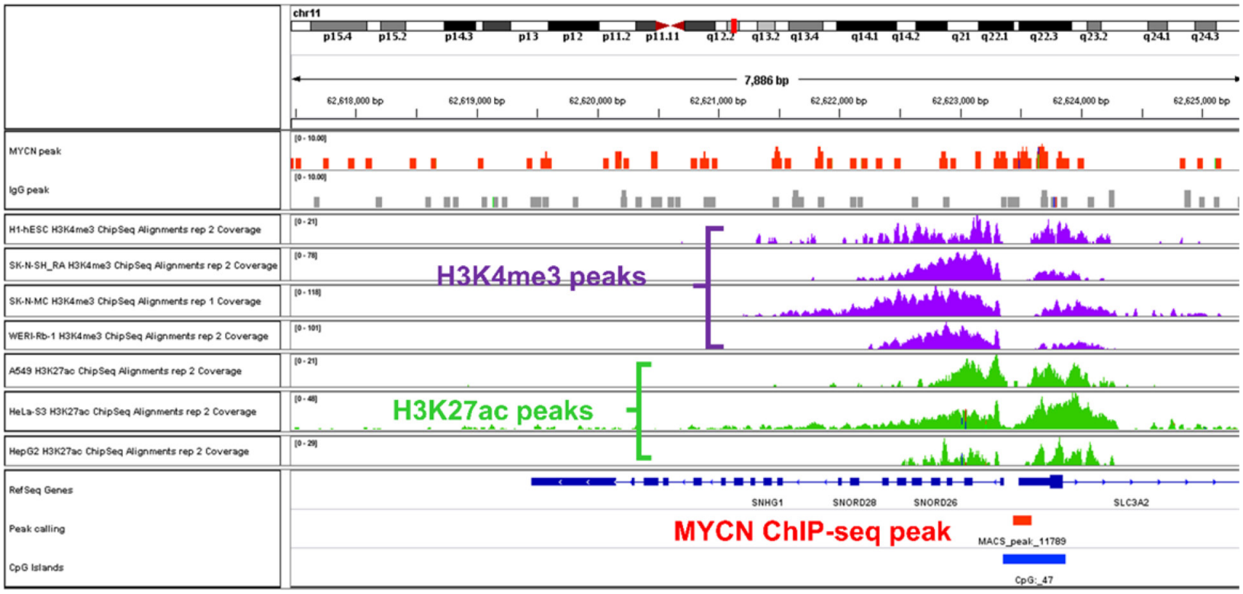

Supplementary Figure S5: A graphical representation showing MYCN binding sites at -230 bp to -79 bp upstream of *SNHG1* transcription start site (MACS_peak_11789) in SK-N-BE(2)-C cell line. Here, histone modification (H3K4me3 peaks and H3K27ac peaks) and CpG island (CpG islands peaks) are promoter region marker, loaded from the ENCODE database and shown with purple, green and blue color respectively. Chip-seq data visualized using Integrative Genomics Viewer.

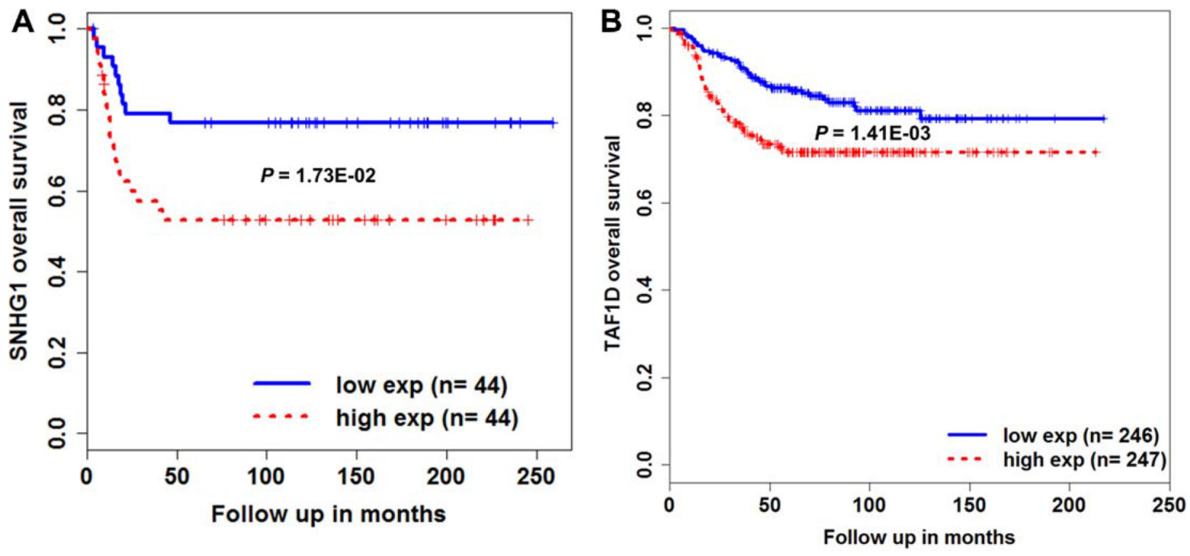

Supplementary Figure S6: (**A**, **B**) Kaplan – Meier curve indicating overall survival for the low-expression versus the high-expression group based on the median expression of *SNHG1* (*n* = 88) and *TAF1D* **(***n* = 493). The *P*-value obtained for the differences in the curve using log rank (Mantel-Haenszel) test.

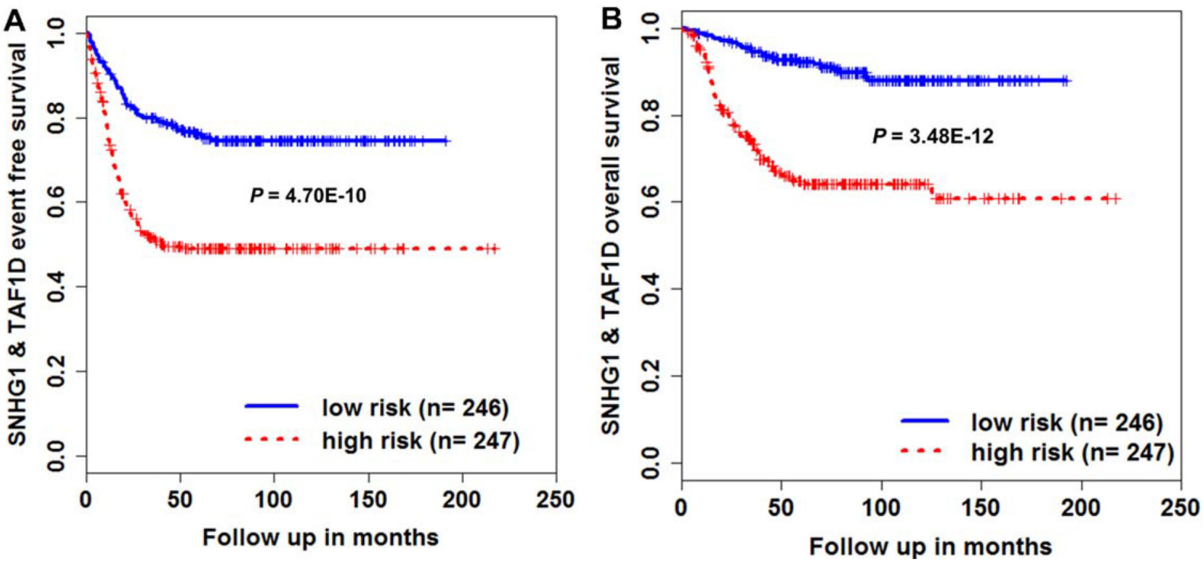

Supplementary Figure S7: (**A**, **B**) Kaplan – Meier curve indicating event-free survival and overall survival for the low-risk versus the high-risk group based on the median risk score. The *P*-value obtained for the differences in the curve using log rank (Mantel-Haenszel) test.

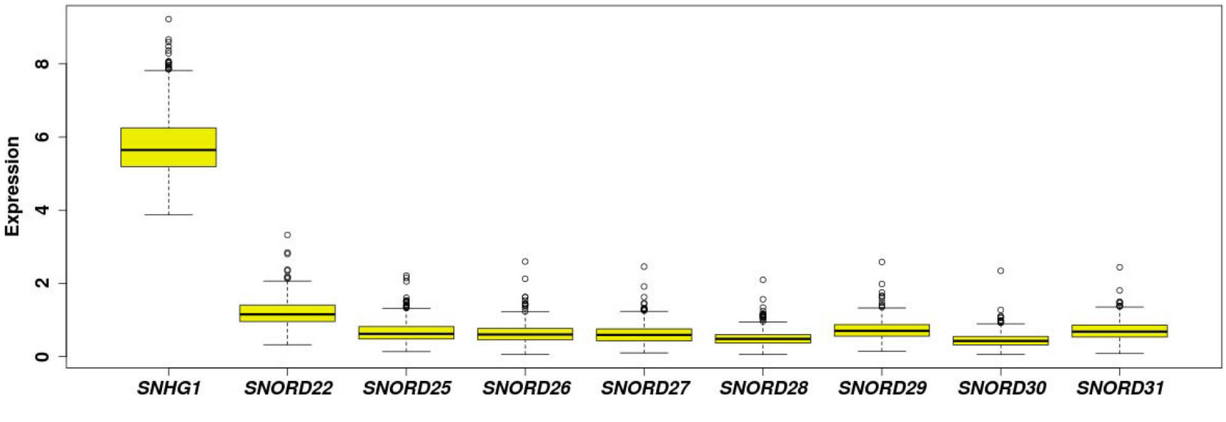

Supplementary Figure S8: Boxplot showing the expression value of *SNHG1* and its small nucleolar RNAs (snors) from the RNA-seq dataset (*n* = 493).

Supplementary Table S1A: Differentially expressed lncRNAs in *MYCN* amplified compared with *MYCN* non-amplified subtype condition in GSE12460 (*n* = 47)

Probe ID	Gene symbol	Fold change	Adjusted *P* value	Regulation
207028_at	*MYCNOS*	5.55	8.09E-12	up-regulation
233562_at	*LINC00839*	4.67	3.42E-04	up-regulation
242546_at	*AL589743.1*	3.19	3.23E-02	up-regulation
229715_at	*RP1-239B22.5*	3.00	4.36E-03	up-regulation
1558819_at	*RP11-159H10.3*	2.62	3.42E-04	up-regulation
224610_at	*SNHG1*	2.47	2.05E-02	up-regulation
227517_s_at	*GAS5*	2.43	2.30E-03	up-regulation
224870_at	*DANCR*	2.34	1.96E-03	up-regulation
224603_at	*SNHG16*	2.23	3.02E-04	up-regulation
1560579_s_at	*RP11-31F15.1*	2.18	3.02E-04	up-regulation
225786_at	*HNRNPU-AS1*	2.03	3.76E-02	up-regulation
237737_at	*RP11-782C8.2*	2.01	3.23E-02	up-regulation
235497_at	*RP11-206L10.11*	0.47	1.45E-03	down-regulation
239742_at	*RP11-732M18.3*	0.46	3.15E-02	down-regulation
243904_at	*RP11-361F15.2*	0.45	4.24E-03	down-regulation
1556195_a_at	*RP4-798A10.7*	0.44	3.76E-02	down-regulation
230737_s_at	*PTCHD3P1*	0.40	1.96E-03	down-regulation
226582_at	*RP11-834C11.4*	0.39	2.16E-03	down-regulation
227909_at	*LINC00086*	0.38	2.58E-02	down-regulation
213158_at	*RP11-553L6.5*	0.33	1.19E-03	down-regulation
1560692_at	*GS1-18A18.1*	0.28	1.20E-02	down-regulation

Supplementary Table S1B: Differentially expressed mRNAs in *MYCN* amplified compared with *MYCN* non-amplified subtype condition in GSE12460 (*n* = 47). See Supplementary_Table_S1B

Supplementary Table S2A: Differentially expressed lncRNAs in *MYCN* amplified compared with *MYCN* non-amplified subtype condition in GSE62564 (*n* = 493). See Supplementary_Table_S2A

Supplemenatary Table S2B: Differentially expressed mRNAs in *MYCN* amplified compared with *MYCN* non-amplified subtype condition in GSE62564 (*n* = 493). See Supplementary_Table_S2B

Supplementary Table S3: Multivariate Cox-regression analysis for combinatorial effect of *SNHG1* & *TAF1D* with established factors in GSE62564 NB cohort

Variables	Event-free survival		
	HR	95% CI	*P*
MYCN (amp vs non-amp)	1.51	(1.05–2.15)	2.54E-02
Stage *(4 vs 1,2,3 & 4S)*	2.01	(1.38–2.94)	2.99E-04
Age *(≥ 18 months vs < 18 months)*	1.95	(1.37–2.78)	2.02E-04
SNHG1 exp (≥ 5.65 vs < 5.65)	**1.65**	(1.12–2.43)	**1.18E-02**
MYCN (amp vs non-amp)	1.34	(0.92–1.94)	1.30E-01
Stage *(4 vs 1,2,3 & 4S)*	2.12	(1.44–3.11)	1.29E-04
Age *(≥ 18 months vs < 18 months)*	1.98	(1.39–2.82)	1.59E-04
SNHG1 (≥ 5.65 vs < 5.65)	1.22	(0.73–2.02)	4.44E-01
SNHG1 & TAF1D risk score (≥ 5.02 vs < 5.02)	**1.56**	(0.97–2.5)	**6.54E-02**
MYCN (amp vs non-amp)	1.33	(0.9–1.98)	1.55E-01
Stage *(4 vs 1,2,3 & 4S)*	2.25	(1.56–3.25)	1.46E-05
Age *(≥ 18 months vs < 18 months)*	1.98	(1.39–2.82)	1.61E-04
TAF1D (≥ 4.11 vs < 4.11)	1.05	(0.71–1.55)	8.02E-01
SNHG1 & TAF1D risk score (≥ 5.02 vs < 5.02)	**1.72**	(1.14–2.58)	**9.26E-03**

Supplementary Table S4: Cox proportional hazard analysis of EFS in NB

Variable	rho	chisq	*P* value
MYCN (amp vs non-amp)	–0.03	0.15	7.02E-01
SNHG1 (≥ 5.65 vs < 5.65)	–0.04	0.42	5.18E-01
TAF1D (≥ 4.11 vs < 4.11)	–0.02	0.05	8.30E-01
Stage *(4 vs 1,2,3 & 4S)*	0.16	5.52	1.88E-02
Age *(≥ 18 months vs < 18 months)*	0.34	20.66	5.48E-06
GLOBAL	NA	46.33	7.79E-09

Supplementary Table S5: The sequences of primers for each lncRNA used in RT-qPCR study

lncRNA	Forward	Reverse
MYCNOS	TCCGACAGCTCAAACACAGAC	CCAGCTTTGCAGCCTTCTC
LINC00839	AGCTGGTCTCATCAAGTGGC	GGTTTTGGCCAAGGGATTGC
SNHG1	TAACCTGCTTGGCTCAAAGGG	CAGCCTGGAGTGAACACAGA
DANCR	AGCTCCAGGAGTTCGTCTCTT	AGCTTCAATGGCTTGTGCCT
GAS5	CTTCTGGGCTCAAGTGATCCT	TTGTGCCATGAGACTCCATCAG
SNHG16	CAGTCAGCCTCAGTTTCCAA	AGGCAGGGCTGTGCTGAT
TAF1D	CCATGTGACATCTGATGCTGTG	CCCCTTTAGGTGAGTAAGGGATAC
MYCN	AGCGCCGCAACGACCTTC	CTTGGCGGCCTTCTCATTCTTT
GAPDH	ACACCCACTCCTCCACCTTTG	GCTGTAGCCAAATTCGTTGTCATAC
